# Supplementary material for: Identification and functional analysis of non-coding regulatory small RNA FenSr3 in Bacillus amyloliquefaciens LPB-18
Source: PeerJ. 2023 May 15;11:e15236. doi: 10.7717/peerj.15236 (PMC10194069; doi:10.7717/peerj.15236)
Supplement: Supplemental Information 4 [file peerj-11-15236-s004.zip › KO/CK-vs-T1_map/map04658.html]

KEGG PATHWAY: Th1 and Th2 cell differentiation - Reference pathway


|  |  |
| --- | --- |
| **Th1 and Th2 cell differentiation - Reference pathway** |  |

[
Pathway menu
| Organism menu
| Pathway entry
| Show description
| User data mapping
]

Immunity to different classes of microorganisms is orchestrated by separate lineages of effector T helper (TH)-cells, which differentiate from naive CD4+ precursor cells in response to cues provided by antigen presenting cells (APC) and include T helper type 1 (Th1) and Th2. Th1 cells are characterized by the transcription factor T-bet and signal transducer and activator of transcription (STAT) 4, and the production of IFN-gamma. These cells stimulate strong cell-mediated immune responses, particularly against intracellular pathogens. On the other hand, transcription factors like GATA-3 and STAT6 drive the generation of Th2 cells that produce IL-4, IL-5 and IL-13 and are necessary for inducing the humoral response to combat parasitic helminths (type 2 immunity) and isotype switching to IgG1 and IgE. The balance between Th1/Th2 subsets determines the susceptibility to disease states, where the improper development of Th2 cells can lead to allergy, while an overactive Th1 response can lead to autoimmunity.

|  |  |  |
| --- | --- | --- |
| Reference pathway | 184% 150% 122% 100% 82% 67% 55% | 图片下载 |
